# Piceatannol Alleviates Deoxynivalenol-Induced Damage in Intestinal Epithelial Cells via Inhibition of the NF-κB Pathway

**DOI:** 10.3390/molecules29040855

**Published:** 2024-02-14

**Authors:** Min Zhu, En-Qing Lu, Yong-Xia Fang, Guo-Wei Liu, Yu-Jie Cheng, Ke Huang, E Xu, Yi-Yu Zhang, Xiao-Jing Wang

**Affiliations:** 1 Key Laboratory of Animal Genetics, Breeding and Reproduction in the Plateau Mountainous Region, Ministry of Education, College of Animal Science, Guizhou University, Guiyang 550025, China; enlugzu0622@163.com (E.-Q.L.); 18708634765@163.com (Y.-X.F.); gwliugzu163@163.com (G.-W.L.); 18285695928@163.com (Y.-J.C.); 18884990402@163.com (K.H.); exu@gzu.edu.cn (E.X.); zyy8yyc@163.com (Y.-Y.Z.); 2 Institute of Animal Nutrition and Feed Science, Guizhou University, Guiyang 550025, China; 3 Key Laboratory of Plant Resource Conservation and Germplasm Innovation in Mountainous Region, Ministry of Education, Institute of Agro-Bioengineering, Guizhou University, Guiyang 550025, China

**Keywords:** piceatannol, deoxynivalenol, oxidative stress, NF-κB pathway, intestinal barrier

## Abstract

Deoxynivalenol (DON) is a common mycotoxin that is widely found in various foods and feeds, posing a potential threat to human and animal health. This study aimed to investigate the protective effect of the natural polyphenol piceatannol (PIC) against DON-induced damage in porcine intestinal epithelial cells (IPEC-J2 cells) and the underlying mechanism. The results showed that PIC promotes IPEC-J2 cell proliferation in a dose-dependent manner. Moreover, it not only significantly relieved DON-induced decreases in cell viability and proliferation but also reduced intracellular reactive oxygen species (ROS) production. Further studies demonstrated that PIC alleviated DON-induced oxidative stress damage by increasing the protein expression levels of the antioxidant factors NAD(P)H quinone oxidoreductase-1 (NQO1) and glutamate–cysteine ligase modifier subunit (GCLM), and the mRNA expression of *catalase* (*CAT*), *Superoxide Dismutase 1* (*SOD1*), *peroxiredoxin 3* (*PRX3*), and *glutathione S-transferase alpha 4* (*GSTα4*). In addition, PIC inhibited the activation of the nuclear factor-B (NF-κB) pathway, downregulated the mRNA expression of interleukin-1β (IL-1β), interleukin-6 (IL-6), and tumor necrosis factor α (TNF-α) to attenuate DON-induced inflammatory responses, and further mitigated DON-induced cellular intestinal barrier injury by regulating the protein expression of Occludin. These findings indicated that PIC had a significant protective effect against DON-induced damage. This study provides more understanding to support PIC as a feed additive for pig production.

## 1. Introduction

Deoxynivalenol (DON) is a trichothecene mycotoxin that is mainly produced by *Fusarium graminearum* and *Fusarium flavum* and is typically found in feed and other organic substrates [1]. Because DON can cause vomiting in pigs, it is also known as a vomiting toxin. As one of the most commonly detected toxins in the world, DON pollution is widespread worldwide. Given that DON is ubiquitous in human and animal food, it poses a potential risk to human and animal health [2]. Although exposure to DON could result in its accumulation in animal-derived products, such as muscle, the amount that consumers are exposed to through consumption is very low [3]. DON is classified in Group 3 (not classifiable for its carcinogenicity to humans) by the International Agency for Research on Cancer (IARC), a division of the World Health Organization (WHO) [4]. When humans or animals are exposed to DON, the body will experience a series of adverse reactions, such as anorexia, vomiting, diarrhea, intestinal inflammation, and immune system diseases, which seriously endanger health [5,6]. Studies have shown that when cells are exposed to DON, cell viability and antioxidant capacity decrease, leading to inflammation and apoptosis [7]. As previously mentioned, it is imperative to develop effective methods to destroy or inactivate DON in food and feed. Currently, common methods for detoxifying DON include physical, chemical, and biological techniques, such as heat treatment, ozonization, and the use of microbes or enzymes [8].

Compared with other monogastric farm animals, pigs are more sensitive to DON [9]. The intestinal epithelium is the main barrier of the body, allowing the absorption of nutrients while preventing pathogenic microorganisms and toxins from entering the body [10]. Therefore, after the ingestion of infected feed, intestinal epithelial cells are exposed to high concentrations of DON, which may cause a variety of intestinal diseases [11]. The IPEC-J2 cell line is a well-established in vitro model of intestinal physiology [12], and it is often used for in vitro toxicity studies, barrier regulation, nutritional uptake, and drug function screening [13,14]. It has been reported that when IPEC-J2 cells are exposed to DON, cell viability is reduced and cell barrier integrity is damaged [7,15]. DON can also interfere with cell cycle progression from the G2/M phase to the S phase, thereby affecting cell proliferation, and can regulate the expression levels of factors associated with barrier function, nutrient transport, and mitochondrial biosynthesis and function-related genes [16]. Therefore, there is an urgent need to identify effective ways to relieve DON-induced cytotoxicity to reduce its potential risks to animal and human health.

Passion fruit (*Passiflora edulis*) seeds contain considerable quantities of a natural polyphenolic compound called piceatannol (PIC) [17]. PIC is also present in red grapes and other plants [18]. PIC is similar to resveratrol (RES) and shares similar biological activities. It has been shown to possess various beneficial properties, including antioxidation, anti-inflammation, and neuroprotective activities [19,20,21]. Li’s research demonstrated that PIC could suppress inflammation induced by tumor necrosis factor-alpha (TNF-α) [22]. A study revealed that PIC could inhibit the production of PGE2 and NO induced by lipopolysaccharide. This is achieved by regulating the expression of the transcription factor nuclear factor kappa-B (NF-κB) in RAW 264.7 macrophages [23]. In addition, PIC has been reported to protect ARPE-19 cells against photooxidative damage caused by vitamin A dimers, which is achieved by activating the nuclear factor erythroid 2-related factor-2 (Nrf2)/NAD(P)H quinone oxidoreductase-1 (NQO1) signaling pathway [24]. Recent research has shown that PIC can reduce aging by activating Nrf2 and its downstream antioxidant enzyme in the cranial nerve [25]. However, whether PIC can alleviate DON-induced IPEC-J2 cytotoxicity has not been reported, and the protective effect of PIC on IPEC-J2 cells is also unclear.

Therefore, in this study, IPEC-J2 cells were used as a model to investigate the protective effect of PIC against DON-induced IPEC-J2 cell injury and the possible molecular mechanism. This study provides a theoretical basis for the use of PIC as a feed additive to maintain porcine intestinal health.

## 2. Results

### 2.1. Effects of PIC on the Viability of IPEC-J2 Cells

**Figure 1:**
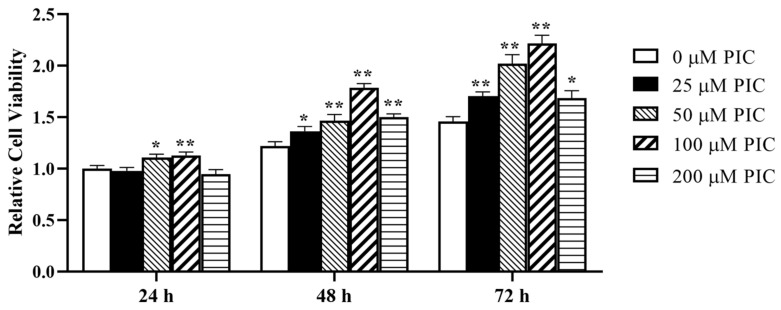
Effects of different concentrations of PIC on the viability of IPEC-J2 cells. Cell viability was determined at 24 h, 48 h, and 72 h (*n* = 6). The data are means ± SEMs. Note: ** means *p* < 0.01 and * means *p* < 0.05 compared with the control group (0 μM PIC).

IPEC-J2 cells were incubated with PIC (0, 25, 50, 100, and 200 μM) for 24 h, 48 h, and 72 h. As shown in Figure 1, 50 or 100 μM PIC treatment for 24 h could enhance cell viability (*p* < 0.05), and 100 μM PIC was preferable. Treatment with 25–200 μM PIC for 48 h or 72 h could stimulate cell viability (*p* < 0.01), and 100 μM PIC was the most effective. Therefore, 100 μM PIC was chosen for the next experiment.

### 2.2. PIC Alleviates the Cytotoxicity of IPEC-J2 Cells Induced by DON

**Figure 2:**
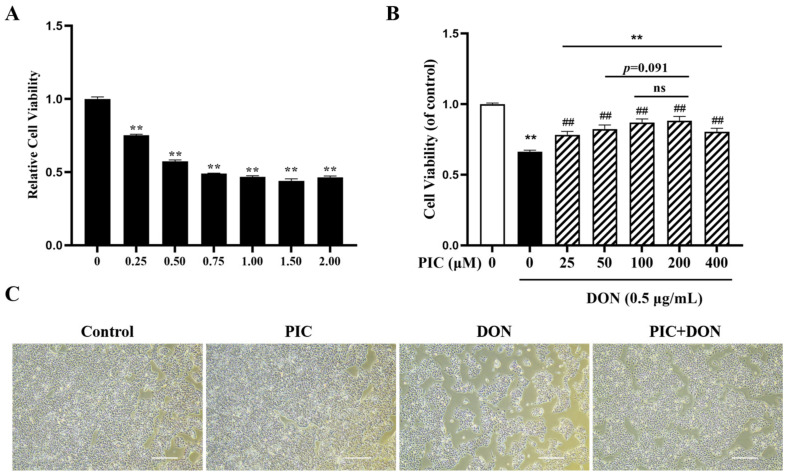
PIC attenuated DON-induced cytotoxicity in IPEC-J2 cells. (**A**) Cells were treated with 0, 0.25, 0.5, 0.75, 1, 1.5, and 2 μg/mL DON for 24 h (*n* = 6). (**B**) Cells were pretreated for 24 h with PIC (0, 25, 50, 100, 200, and 400 μM) before being incubated with DON (0.5 μg/mL) for another 24 h. (**C**) Morphological observations of CON-, PIC-, DON-, and PIC + DON-treated IPEC-J2 cells (4× magnification). The data are means ± SEMs. Note: ** means *p* < 0.01 compared with the control group; ## means *p* < 0.01 compared with the DON alone group. “ns” means non-statistically significant.

IPEC-J2 cells were exposed to several doses of DON (0, 0.25, 0.5, 0.75, 1, 1.5, and 2 μg/mL) for 24 h, and DON showed significant cytotoxicity. As shown in Figure 2A, in response to 0.5 μg/mL DON, cell viability was 57.34% (*p* < 0.01), which is similar to the IC50 of DON in IPEC-J2 cells.

IPEC-J2 cells were pretreated with 0, 25, 50, 100, 200, and 400 µM PIC for 24 h before being treated with 0.5 μg/mL DON for 24 h. As expected, PIC could attenuate DON-induced cytotoxicity. Compared with that in the control group, cell viability in the DON group was significantly decreased (*p* < 0.01). However, after pretreatment with 25–400 μM PIC for 24 h, cell viability was significantly increased compared with that in the DON-only group (*p* < 0.01, Figure 2B). It is worth noting that when the PIC concentration was 100 μM and 200 μM, cell viability was restored to the highest level, and there was no difference between the two groups (Figure 2B). Thus, 100 µM PIC was selected as the concentration for subsequent experiments. Cell morphology was further examined and showed that DON resulted in a flattened cell shape and reduced cell numbers. Pretreatment with PIC significantly reversed these cellular morphological changes (Figure 2C).

### 2.3. Effects of DON and PIC on IPEC-J2 Cell Growth

**Figure 3:**
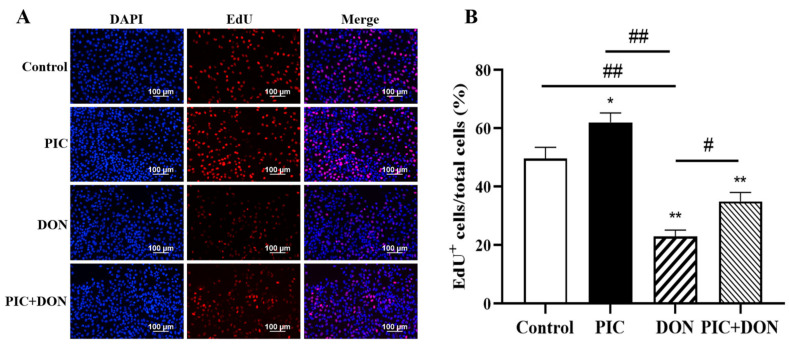
Effects of PIC and DON on the proliferation of IPEC-J2 cells. Cells were pretreated with PIC (100 μM) for 24 h and then exposed to DON (0.5 μg/mL) for 24 h. EdU incorporation was used to measure cell proliferation (*n* = 6). (**A**) Images of EdU-positive cells (red nuclei) at 200× magnification. (**B**) Cell proliferation is presented as the percentage of cells (blue nuclei) that were EdU-positive (red nuclei). The data are means ± SEMs. Note: ** means *p* < 0.01 and * means *p* < 0.05 compared with the control group; ## means *p* < 0.01 and # means *p* < 0.05 compared with the DON-alone group.

The proliferation of cells in the S phase of the cell cycle can be assessed by using 5-Ethynyl-2′-deoxyuridine (EdU) to measure the proliferative capacity of IPEC-J2 cells. The proliferating cells in which the nucleus was stained red were considered EdU-positive cells. As shown in Figure 3A,B, the percentage of EdU-positive cells in the DON-treated group was considerably lower (*p* < 0.01) than that in the control group. Importantly, PIC could increase IPEC-J2 cell proliferation. After PIC pretreatment, the number of EdU-positive cells increased considerably in the PIC+DON group compared with the DON group (*p* < 0.01). This finding suggests that PIC pretreatment can enhance the proportion of EdU-positive cells following DON injury, thereby reducing the effects of DON.

### 2.4. PIC Eliminates DON-Induced Reactive Oxygen Species (ROS) Production

**Figure 4:**
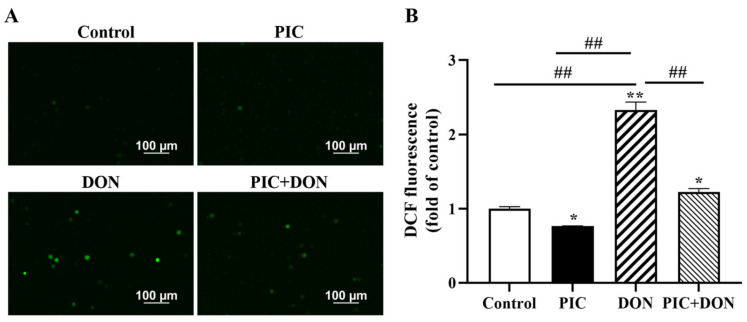
PIC decreased DON-induced ROS generation in IPEC-J2 cells. Cells were pretreated with PIC (100 μM) for 24 h and then exposed to DON (0.5 μg/mL) for 24 h. (**A**) After being incubated with 2′, 7′-Dichlorofluorescin diacetate (DCFH-DA), the cells were examined with a fluorescence microscope (scale bar: 100 μm). (**B**) The fluorescence intensity of ROS in IPEC-J2 cells was analyzed by ImageJ software (Version 1.8.0 112, National Institutes of Health, Bethesda, MD, USA). The data are means ± SEMs. Note: ** means *p* < 0.01 and * means *p* < 0.05 compared with the control group; ## means *p* < 0.01 compared with the DON alone group.

As shown in Figure 4, the PIC group had dramatically reduced intracellular ROS levels (*p* < 0.05) compared with the control group. Intracellular ROS levels increased considerably (*p* < 0.01) after DON stimulation. PIC pretreatment significantly reduced ROS generation (*p* < 0.01). This finding demonstrates that PIC can reduce DON-induced ROS generation, thereby protecting cells from free radical damage.

### 2.5. PIC Protected IPEC-J2 Cells from DON-Induced Oxidative Damage

**Figure 5:**
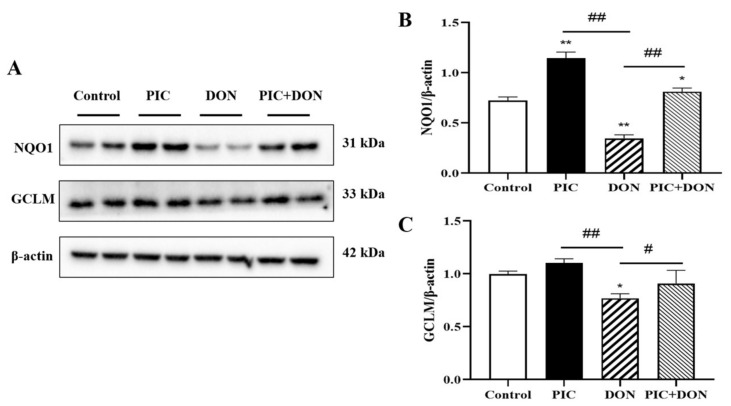
The effect of PIC on the expression of antioxidant proteins in DON-treated IPEC-J2 cells. Cells were pretreated with PIC (100 μM) and then exposed to DON (0.5 μg/mL) for 24 h. (**A**) The expression levels of NQO1 and GCLM proteins were detected by Western blotting, and β-Actin served as an internal control. (**B**) The NQO1/β-Actin ratio. (**C**) The GCLM/β-Actin ratio. The data are means ± SEMs (*n* = 3). Note: ** means *p* < 0.01 and * means *p* < 0.05 compared with the control group; ## means *p* < 0.01, # means *p* < 0.05, compared with the DON-alone group.

Western blotting was used to determine the protein expression levels of NQO1 and glutamate–cysteine ligase modifier subunit (GCLM) to investigate the effect of PIC on DON-induced oxidative damage in IPEC-J2 cells. These two proteins play critical antioxidant and detoxifying roles in cells, ensuring intracellular redox homeostasis and protecting cells from oxidative damage. As shown in Figure 5, the protein expression levels of NQO1 and GCLM in the DON group were considerably lower than those in the control group (*p* < 0.05). Compared with the DON group, PIC pretreatment significantly elevated NQO1 and GCLM protein expression (*p* < 0.05). Other popular antioxidant genes, namely, *catalase* (*CAT*), *Heme Oxygenase-1* (*HO-1*), *Superoxide Dismutase 1* (*SOD1*), *peroxiredoxin 1* (*PRX1*), *PRX3*, *glutathione peroxidases 3* (*GPX3*), *GPX4*, *glutathione S-transferase alpha 1* (*GSTα1*), and *GSTα4*, were measured by RT-qPCR as well (refer to Figure S1). Compared with the control group, DON reduced the gene expression of *CAT*, *SOD1*, *PRX3*, *GPX3*, *GPX4*, *GSTα1*, and *GSTα4*, but no change was observed in *HO-1* and *PRX1*. Additionally, PIC pretreatment increased the mRNA expression of *CAT*, *SOD1*, *PRX3*, and *GSTα4* when compared with the DON group. These suggest that PIC can increase antioxidant capability and reduce the oxidative damage caused by DON in IPEC-J2 cells.

### 2.6. PIC Reduced DON-Induced Inflammation in IPEC-J2 Cells

**Figure 6:**
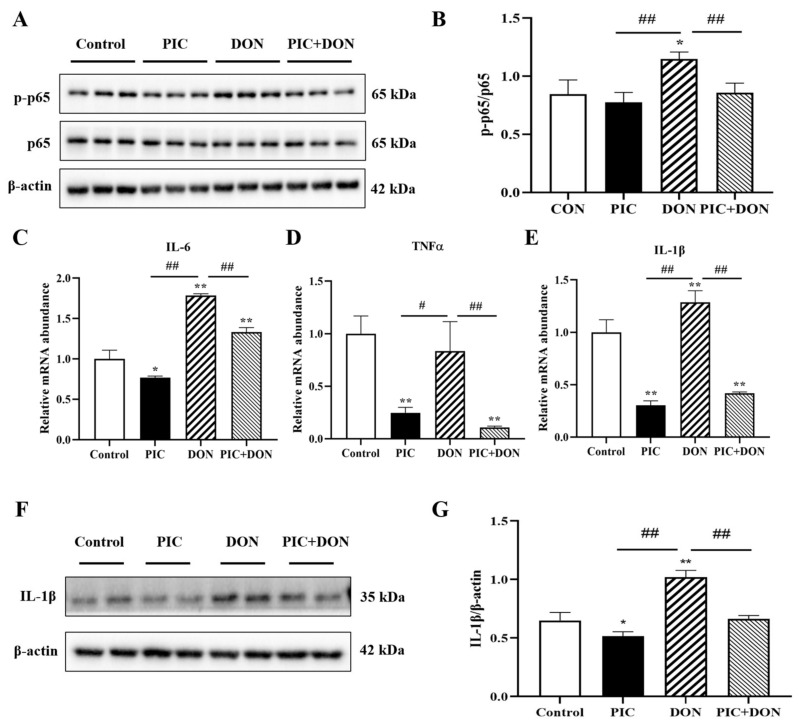
The effect of PIC on DON-induced mRNA and protein expression of inflammatory factors in IPEC-J2 cells. Cells were pretreated with PIC (100 μM) and then exposed to DON (0.5 μg/mL) for 24 h. (**A**) The protein expression of p-p65 and p65 was detected by Western blotting. (**B**) The p-p65/p65 ratio. (**C**–**E**) The mRNA expression of IL-6, TNFα, and IL-1β. (**F**) The protein expression of IL-1β. (**G**) The IL-1β/β-Actin ratio. The data are means ± SEMs (*n* = 3). Note: ** means *p* < 0.01 and * means *p* < 0.05 compared with the control group; ## means *p* < 0.01 and # means *p* < 0.05 compared with the DON-alone group.

The activity of the NF-κB signaling pathway was detected. As shown in Figure 6, compared with the control group, the DON treatment group had significant increases in the mRNA expression of IL-6 and IL-1β (*p* < 0.01), the protein expression of IL-1β (*p* < 0.01), and the ratio of p-p65/p65 (*p* < 0.05). Interestingly, PIC pretreatment inhibited DON-induced increases in IL-6, TNF-α, and IL-1β mRNA expression (*p* < 0.01), IL-1β protein expression (*p* < 0.01), and the p-p65/p65 ratio (*p* < 0.01). This shows that PIC can reduce DON-induced damage to cells and reduce the occurrence of the inflammatory response.

### 2.7. PIC Regulated the Expression of DON-Induced Tight Junction Proteins in IPEC-J2 Cells

**Figure 7:**
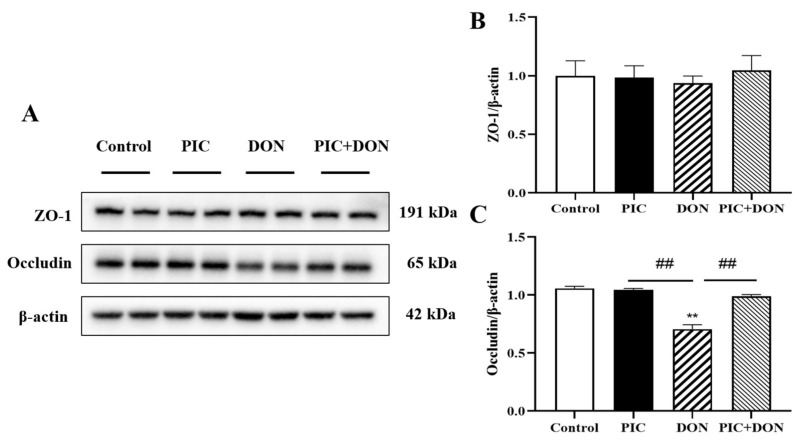
Effect of PIC on DON-induced changes in tight junction proteins in IPEC-J2 cells. Cells were pretreated with PIC (100 μM) and then exposed to DON (0.5 μg/mL) for 24 h. (**A**) The protein expression of ZO-1 and Occludin was detected by Western blotting, and β-Actin served as an internal control. (**B**) The ZO-1/β-Actin ratio. (**C**) The Occludin/β-Actin ratio. The data are means ± SEMs (*n* = 3). Note: ** means *p* < 0.01 compared with the control group; ## means *p* < 0.01 compared with the DON-alone group.

We performed Western blotting to measure the protein expression levels of ZO-1 and Occludin to investigate the impact of PIC and DON on tight junction proteins. As shown in Figure 7, compared with that in the control group, the protein expression level of Occludin in IPEC-J2 cells treated with DON alone was significantly decreased (*p* < 0.05). In addition, compared with that in the DON-treated group, the protein expression of Occludin in PIC-pretreated cells was significantly increased (*p* < 0.01). However, ZO-1 expression was not changed in any of the groups. Overall, these results indicated that PIC pretreatment could enhance the tight junctions between intestinal epithelial cells and protect IPEC-J2 cells from DON injury.

## 3. Discussion

Pigs are one of the most sensitive farmed animals to DON [26]. During the breeding process, DON is mainly absorbed in the small intestine [27]. Early results suggest that the presence of DON could cause inflammation and apoptosis, break down barrier integrity, affect the structure of the intestine, and decrease the efficiency of nutrient absorption and transport in pigs [15,28,29]. Therefore, it is crucial to develop effective strategies to mitigate the toxic impact of DON on intestinal epithelial cells.

Plant polyphenols are a class of compounds that are widely found in nature. Due to their unique chemical properties and structure, plant polyphenols have various biological effects, including antioxidant and anti-inflammatory effects, can inhibit diabetes and obesity, and can prevent and treat cardio- and cerebrovascular diseases [30,31,32]. Many researchers have used polyphenols to detoxify mycotoxins. Studies have shown that astaxanthin can decrease the impact of aflatoxin B1 on oxidative stress and apoptosis in IPEC-J2 cells [33]. Similarly, RES protects IPEC-J2 cells from DON-induced injury through the Nrf2 signaling pathway [34]. Based on this, we hypothesized that PIC, which is a phenolic compound, may also influence mycotoxin toxicity. To confirm this hypothesis, we investigated the effect of PIC on DON-induced cytotoxicity by examining oxidative stress, inflammation, and the intestinal barrier using the IPEC-J2 cell line.

In this study, we observed that the viability of IPEC-J2 cells decreased gradually with increasing DON concentrations, which is consistent with previous findings [7]. After treatment with 0.5 μg/mL DON for 24 h, cell viability decreased to approximately 57%. When the cells were treated with 50 μM and 100 μM PIC for 24 h, the viability of IPEC-J2 cells was increased, and the effect of 100 μM PIC was better. Similar to our study, rat cardiomyocytes were treated with 1–50 μM PIC, which increased cell viability [35]. It has also been reported that when human retinal pigment epithelial cells were exposed to hydrogen peroxide (H_2_O_2_), cell viability was reduced, and in response to supplementation with 5–15 µM PIC, cell viability gradually recovered to its original level [36]. In our study, PIC alleviated DON-induced cytotoxicity and increased cell viability from 62% to 85%. The results of EdU staining were consistent with this finding. The results showed that PIC protected cells by inhibiting cell death and improving cell growth.

An imbalance between prooxidants and antioxidants can lead to oxidative stress, which destroys the intestinal barrier and promotes apoptosis, leading to intestinal diseases [37,38]. Excessive ROS or antioxidant deficiencies often lead to oxidative damage and cell mortality [39]. Earlier studies showed that DON increases ROS levels in IPEC-J2 cells and induces cell death [40]. PIC is a potent ROS scavenger that protects cells from ROS accumulation and oxidative damage-induced apoptosis [36]. Our research indicated that PIC pretreatment significantly inhibited DON-induced ROS accumulation. Nrf2 is a central transcription factor that encodes antioxidant proteins and detoxification enzymes to combat oxidative damage [41]. It is well known that SOD1, CAT, HO-1, PRX1, PRX3, GPX3, GPX4, GSTα1, GSTα4, GCLM, and NQO1 are target genes of Nrf2 [42,43,44]. SOD converts superoxide anions to H_2_O_2_, and CAT breaks down H_2_O_2_ into O_2_ and H_2_O. PRXs reduce ROS, and GPXs regulate cytoplasmic H_2_O_2_ levels. GST uses glutathione to detoxify xenobiotics and their reactive metabolites, while HO-1 is an enzyme that limits the degradation of heme and prevents free heme from participating in the oxidative reaction. GCLM is the rate-limiting enzyme for glutathione synthesis, while NQO1 protects cells from oxidative stress by regulating cellular redox status [42,43,44]. In this study, it was observed that the mRNA levels of *CAT*, *SOD1*, *PRX3*, and *GSTα4* were significantly reduced in IPEC-J2 cells when exposed to DON. However, when co-treated with PIC, the mRNA levels of these antioxidant genes were significantly increased. There was no change in the mRNA expression of *HO-1*, indicating that PIC does not work through this gene. The protein expression levels of intracellular antioxidants NQO1 and GCLM were increased, which helped to reduce DON-induced oxidative damage in IPEC-J2 cells. Overall, these findings suggest that PIC has an antioxidant effect and can protect IPEC-J2 cells from DON-induced damage.

Oxidative damage and inflammation levels are closely related in cells [45]. Therefore, we investigated the effect of PIC on DON-induced NF-κB pathway activation. The results showed that DON promoted p65 phosphorylation and increased the mRNA expression of the inflammatory factors IL-6 and IL-1β and the protein expression of IL-1β, leading to increased cellular inflammation. Encouragingly, PIC pretreatment could alleviate inflammation caused by DON exposure. Similarly, an alkaloid called koumine alleviated LPS-induced toxicity in RAW264.7 macrophages by inhibiting the NF-κB pathway [46]. Additionally, ferulic acid (FA) attenuated DON-induced inflammation in IPEC-J2 cells by inhibiting the phosphorylation of NF-κB [47]. These results indicate that PIC has a good effect on the inflammatory response caused by DON.

Tight junctions are multiprotein complexes that are used to maintain intestinal epithelial barrier function [48]. Occludin and ZO-1 are key tight junction proteins that are major targets in the treatment of intestinal barrier function [49]. It has been reported that reductions in intestinal permeability in weaned piglets caused by a deficiency of tight junction proteins can be ameliorated by upregulating the expression of Occludin and ZO-1 [50]. In this study, we found that exposure to DON resulted in the downregulation of Occludin protein expression, which was consistent with previous research results [30]. However, the protein expression of ZO-1 remained unchanged. After PIC pretreatment, the protein expression of Occludin was upregulated to reduce intestinal permeability. The PIC-induced decrease in intestinal permeability might prevent DON from damaging the intestinal barrier, suggesting that PIC may protect IPEC-J2 cells against DON-induced injury by enhancing the function of intestinal tight junction proteins.

## 4. Materials and Methods

### 4.1. Chemicals and Reagents

IPEC-J2 cells were a gift from the team of Professor Yulong Yin (Chinese Academy of Sciences, Institute of Subtropical Agriculture). Piceatannol (purity ≥ 99%, HY-132179, MCE), Cell Counting Kits (CCK-8) (K1018, APExBIO, Houston, TX, USA), EdU enhancement detection kits (C10310, RIBOBIO, Guangdong, China), and ROS detection reagent (S0033S, Beyotime, Shanghai, China) were used. High-glucose DMEM (C11995500BT, Gibco, Waltham, MA, USA), fetal bovine serum (FBS, C04001, VivaCell, Shanghai, China), and penicillin and streptomycin (03-031-1BCS, BI, Beit HaEmek, Israel) were used. DON (99% purity) and dimethyl sulfoxide (DMSO) were purchased from Sigma-Aldrich. (St. Louis, MO, USA).

### 4.2. Cell Culture

IPEC-J2 cells were grown in high-glucose DMEM containing 10% FBS, 1% penicillin, and streptomycin. When the cells reached 90% confluence, they were passaged.

### 4.3. Cell Treatment

DON was solubilized in DMSO to a concentration of 1 mg/mL. The solution was diluted in the medium indicated for the experiment (0.25, 0.5, 0.75, 1, 1.5, 2 μg/mL). Similarly, PIC was dissolved in DMSO to a concentration of 100 mM and diluted in medium (25, 50, 100, 200 µM) for subsequent experiments. IPEC-J2 cells were treated with different concentrations of PIC and DON for 24 h. Equal amounts of DMSO were added to each test condition.

### 4.4. Cell Viability and Cell Morphology

Cell viability was determined according to the manufacturer’s instructions. Cells were inoculated at 1 × 10^4^ per well into 96-well plates and incubated for 12 h. The medium was discarded, and the cells were incubated with DON at the indicated concentrations for 24 h. PIC was added and incubated for 24, 48, and 72 h. The cells were then placed in a fresh medium containing 10% FBS. A 10% CCK8 solution was added to each well, and the cells were incubated at 37 °C for 2 h. The absorbance of each well was measured at 450 nm using a Bio-Tek microplate reader (SynergyH4, Winooski, VT, USA), and then cell viability was calculated. Cells were seeded in 6-well plates and treated with DON and PIC before cell morphology was observed with an inverted fluorescence microscope (Ti-2, Tokyo, Japan).

### 4.5. Cell Proliferation Assay

The cells were divided into 4 groups: the control group (0.1% DMSO), the PIC group, the DON group, and the PIC+DON group. The control group was treated with a normal medium containing 10% FBS, the PIC group was treated with 100 µM PIC for 24 h, the DON group was treated with 0.5 μg/mL DON for 24 h, and the PIC+DON group was preincubated with 100 µM PIC for 24 h and then treated with 0.5 μg/mL DON for another 24 h. Then, cell proliferation was examined using the Cell-Light EdU kit (RiboBio, Guangzhou, China), and cellular staining was observed under a Nikon microscope (Ti-S, Tokyo, Japan). Three microscopic fields were randomly selected per well for observation. Proliferating cells showed red fluorescence, and nuclei showed blue fluorescence. Cell proliferation was assessed by calculating the number of EdU-positive cells (red nuclei) as a percentage of the total number of cells (blue nuclei).

### 4.6. Intracellular ROS Assay

According to the manufacturer’s guidelines, ROS levels in cells were detected using ROS detection kits (Beyotime, Shanghai, China). After the 4 groups of cells were treated in a six-well plate, the cell culture medium was discarded, and each group of cells was collected using trypsin. Then, the cells were counted and adjusted to the same cell density with PBS, incubated with 10 μM DCDF-DA at 37 °C for 2 h, and washed three times with PBS. The same volume (100 μL) of cell suspension was used for imaging. Images were obtained using a Nikon microscope (Ti-S, Tokyo, Japan). Intracellular ROS levels were quantified by determining the mean fluorescence intensity and were analyzed using ImageJ software (Version 1.8.0 112, National Institutes of Health, Bethesda, MD, USA).

### 4.7. Quantitative Real-Time PCR

After the four groups of cells were treated in six-well plates, total RNA was extracted with an RNA extraction kit (RN001, ES Science, Shanghai, China) according to the manufacturer’s instructions. Then, total RNA was reverse transcribed to cDNA using HifairIII 1st-Strand cDNA Synthesis SuperMix for qPCR (11141ES60, Shanghai Ye Sheng Biotechnology Co., Ltd., Shanghai, China). SYBR Green Master Mix (11202ES08, Yeasun, Shanghai, China) was used to perform fluorescence quantitative PCR. To standardize the results, β-Actin was chosen as the internal reference gene, and the expression levels of the target genes were calculated using the 2^−ΔΔCT^ method. The primers used are shown in Table 1, and the primers (antioxidant genes) are shown in Table S1.

### 4.8. Western Blotting

After the cells were processed in six-well plates, 200 µL of RIPA lysis buffer containing protease inhibitors (CWBIO, Taizhou, China) and phosphatase inhibitors (CWBIO) was added to each well and placed on a 4 °C shaker for 20 min, after which the cells were scraped using a cell scraper and collected into a 1.5 mL centrifuge tube. The cells were then centrifuged at 12,000× *g* for 15 min at 4 °C, and the supernatant was collected and quantified using the BCA method. The protein samples were separated by 10% SDS–PAGE and then transferred to a PVDF membrane. After being blocked in 5% skim milk powder dissolved in TBST for 2 h, the membrane was incubated overnight with primary antibodies on a 4 °C shaking bed. After being washed three times with TBST, the membrane was incubated with secondary antibodies on a room temperature shaking bed for 2 h and then washed three times with TBST. The bands were visualized on a Bio-Rad instrument (Hercules, CA, USA) using an NcmECL Ultra kit (P10200, NCM, Suzhou, China), and finally, the gray values of the bands were analyzed by ImageJ software. Primary antibodies against NQO1, GCLM (glutamate-cysteine ligase regulatory subunit), IL-1β, NF-κB (p65), and p-NF-κB (p-p65) were obtained from ABclonal Technology Co., Ltd. (Woburn, MA, USA). ZO-1 was obtained from Bioss (Woburn, MA, USA), Occludin was obtained from Cell Signaling Technology (Danvers, MA, USA), and β-Actin was obtained from Santa Cruz (Dallas, TX, USA), and they were used at a dilution of 1:1000. HRP-coupled secondary antibodies were used at a dilution of 1:10,000.

### 4.9. Statistical Analysis

All the tests were performed with at least three replicates. The results are presented as the mean ± SEM. The data were statistically analyzed using one-way ANOVA with the least significant difference (LSD) or Duncan’s test by SPSS software (Version 20.0, SPSS Inc., Chicago, IL, USA), and the homogeneity of variance and the normal distribution of variables were accordingly detected by Levene’s test and the Kolmogorov–Smirnov test. Graphs were generated using Graph Pad Prism 8. *p* < 0.05 was regarded as statistically significant.

## 5. Conclusions

In conclusion, our findings indicated that PIC could promote IPEC-J2 cell proliferation; moreover, PIC alleviated the DON-induced decrease in cell viability and improved the morphological changes in cells induced by DON. PIC also reduced intracellular oxidative damage by upregulating the levels of intracellular antioxidant proteins (NQO1, GCLM) and antioxidant genes (CAT, SOD1, PRX3, GSTα4). In addition, we found that PIC inhibited the NF-κB pathway to reduce the mRNA levels of the inflammatory factors IL-6 and IL-1β and IL-1β protein expression, thereby attenuating the inflammatory response in cells. The improvement in intestinal barrier function in cells demonstrated that PIC prevented the aberrant expression of relevant tight junction proteins, suggesting that intestinal barrier function was another critical target by which PIC could mitigate the toxicity of DON. The present study reveals a new way to protect against DON-induced toxicity in vitro and provides new insights to explore the role and mechanism of PIC as an alternative substance to alleviate DON damage.

## Figures and Tables

**Table 1:** Primer sequences of some genes for quantitative real-time PCR.

Genes	Forward Primer (5′-3′)	Reverse Primer (5′-3′)	Product Size (bp)
*IL-6*	AGCAAGGAGGTACTGGCAGA	GTGGTGGCTTTGTCTGGATT	257
*TNFα*	ATGAGCACTGAGAGCATGATC	CGATAACCTCGAAGTGCAGT	169
*IL-1β*	GTTCTCTGAGAAATGGGAGC	CTGGTCATCATCACAGAAGG	143
*β-Actin*	TGCGGGACATCAAGGAGAAG	AGTTGAAGGTAGTTTCGTGG	216

## Data Availability

Data will be made available upon request.

## Supplementary Materials

The following supporting information can be downloaded at: https://www.mdpi.com/article/10.3390/molecules29040855/s1, Figure S1: The effect of PIC on the expression of antioxidant genes in DON-treated IPEC-J2 cells, Table S1: Primers used for quantitative real-time PCR (antioxidant genes).
