# Supplementary material for: Piceatannol Alleviates Deoxynivalenol-Induced Damage in Intestinal Epithelial Cells via Inhibition of the NF-κB Pathway
Source: Molecules. 2024 Feb 14;29(4):855. doi: 10.3390/molecules29040855 (PMC10891758; doi:10.3390/molecules29040855)
Supplement: Supplementary file 1 [file molecules-29-00855-s001.zip › molecules-2830704-supplementary.pdf]

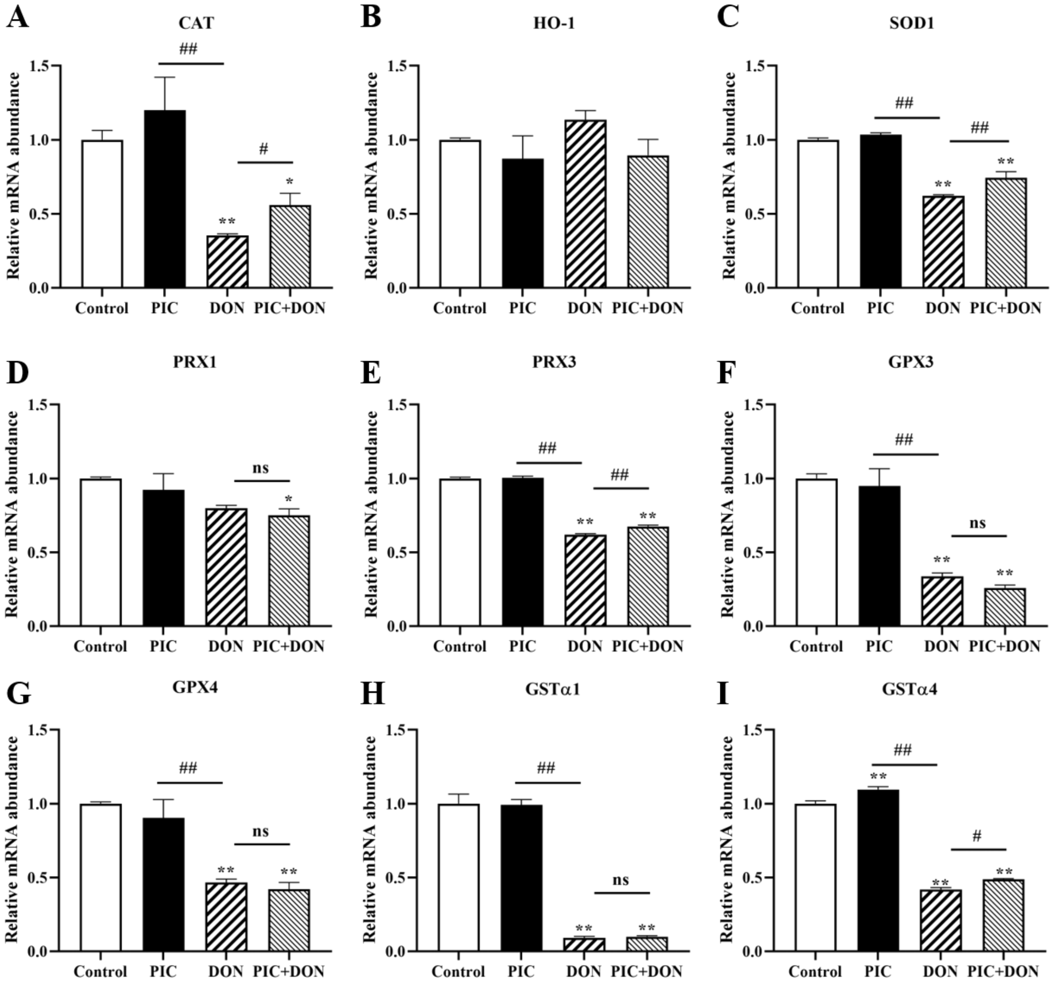

Figure S1. The effect of PIC on the expression of antioxidant genes in DON-treated IPEC-J2 cells. Cells were pretreated with PIC (100 μM) and then exposed to DON (0.5 μg/mL) for 24 h. (**A-I**) The mRNA expression of CAT, HO-1, SOD1, PRX1, PRX3, GPX3, GPX4, GSTα1, GSTα4. The data are means ± SEMs (n=3). Note: ** means $p < 0.01$, and * means $p < 0.05$, compared with the control group; ## means $p < 0.01$, # means $p < 0.05$, compared with the DON alone group. "ns" means none statistic significant.

Table S1. Primers used for quantitative real-time PCR (antioxidant genes).

Genes	Forward Primer (5'-3')	Reverse rimer (5'-3')	Product size (bp)
CAT	TCCAGCCAGTGACCAGATGA	CCCGGTCAAAGTGAGCCATT	182
HO-1	TGATGGCGTCCTTGTACCAC	GACCGGGTTCTCCTTGTTGT	71
SOD1	AAGGCCGTGTGTGTGCTGAA	GATCACCTTCAGCCAGTCCTTT	118
PRX1	GCAGTGAGGACCTTTACCCA	TCACTTTCCTTCCCGATGCC	111
PRX3	GCTGTTAGAAGGTCCCGGTC	GAGGGTCTCTTCCACGCTTC	118
GPX3	GTATGGAGCCCTCACCATCG	TCAGTTCAACGTACTGGCCC	122
GPX4	TGGCCTCTCAATGAGGCAAG	CCCTTGGGCTGGACTTTCAT	253
GSTα1	ACACCCAGGACCAATCTTCTG	AGTCTCAGGTACATTCCGGG	199
GSTα4	CCCCGGATGACATCTACGTG	GGTCCTGTAACACTCACGGG	250
